# Protein-mediated gelation and nano-scale assembly of unfunctionalized hyaluronic acid and chondroitin sulfate

**DOI:** 10.12688/f1000research.16929.3

**Published:** 2019-11-08

**Authors:** Anthony Tabet, June Y. Park, Jarrod Shilts, Kamil Sokolowski, Vijay K. Rana, Marlous Kamp, Nina Warner, Dominique Hoogland, Oren A. Scherman

**Affiliations:** 1 Melville Laboratory for Polymer Synthesis, Department of Chemistry, University of Cambridge, Cambridge, UK; 2 Wellcome Trust Sanger Institute, Hinxton, UK

**Keywords:** hyaluronic acid, chondroitin sulfate, protein-polymer assembly

## Abstract

**Background:** Hyaluronic acid (HA) is a major component of the extracellular matrix (ECM) in the central nervous system and the only purely supramolecular glycosaminoglycan. Much focus has been given to using this high molecular weight polysaccharide for tissue engineering applications. In most studies, the backbone of HA is functionalized with moieties that can facilitate network formation through physical self-assembly, or covalent crosslinking (e.g. photo-catalyzed) at concentrations where the polysaccharide does not gel on its own. However, these crosslinks often utilize functional groups not found in biological tissues.

**Methods: **Oscillatory rheology, dynamic light scattering, and scanning electron microscopy were used to study albumin/HA structures. Dynamic light scattering and transmission electron microscopy were used to study albumin/chondroitin sulfate (CS) structures. UV-vis spectroscopy was used to demonstrate the potential for using protein-polymer blends as an ECM-mimetic model to study transport of small molecules.

**Results: **We examine the intermolecular interactions of two major glycosaminoglycans found in the human brain, HA and the lower molecular weight CS, with the model protein albumin. We report the properties of the resulting micro- and nano materials. Our albumin/HA systems formed gels, and albumin/CS systems formed micro- and nanoparticles. These systems are formed from unfunctionalized polysaccharides, which is an attractive and simple method of forming HA hydrogels and CS nanoparticles. We also summarize the concentrations of HA and CS found in various mammalian brains, which could potentially be useful for biomimetic scaffold development.

**Conclusions: **Simple preparation of commercially available charged biomacromolecules results in interesting materials with structures at the micron and nanometer length-scales. Such materials may have utility in serving as cost-effective models of nervous system electrostatic interactions and as in vitro drug release and model system for ECM transport studies.

## Introduction

**Figure 1.**
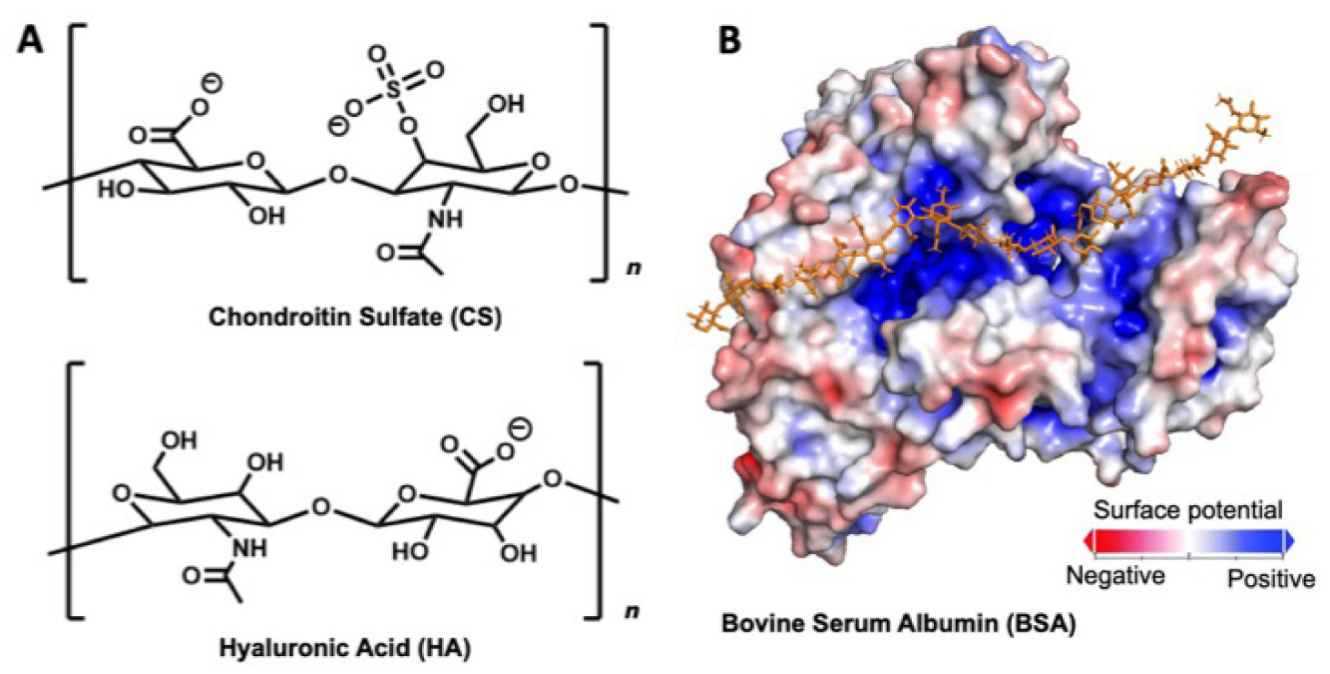
( **A**) Structures of chondroitin sulfate (CS) and hyaluronic acid (HA). ( **B**) Relative surface charge densities of bovine serum albumin (BSA). BSA’s binding pocket is indicated with the example HA chain.

A major paradigm that has dominated the drug delivery and tissue engineering communities is the development of bio-inspired hydrogels that mimic the intermolecular interactions and mechanical properties of physiological tissue. Glycosaminoglycans (GAGs) are polysaccharides that are critical structural components of the brain extracellular matrix (ECM). One particularly abundant brain GAG, hyaluronic acid (HA) ( Figure 1A), has been made into many covalently modified derivatives that have been widely explored in drug delivery and tissue engineering ^1– 3^. Noteably, HA is the only purely supramolecular brain GAG ^4– 8^, *i.e.* weaker and reversible non-covalent interactions dominate over covalent bond formation. Many groups have reported HA- based materials that consist of chemically functionalizing the polymer backbone with moieties that can facilitate formation of supramolecular (such as in physiology) or covalent networks ^9, 10^. Networks formed from unfunctionalized HA, however, have received considerably less attention in the literature. Furthermore, other ECM components, including the more abundant protein-linked GAG chondroitin sulfate (CS) ( Figure 1A), have been comparatively under studied for biomaterial applications ^11^.

In this work we exploit interactions between two major biopolymer components of the brain, HA and CS, with the model protein bovine serum albumin (BSA) ( Figure 1B). Albumin, a major component of blood, has limited permeability through the blood brain barrier (BBB) except in disease cases where the BBB is compromised ^12, 13^. One study showed that microglia, the brain’s resident immune cells, can synthesize albumin, potentially suggesting a more complicated role for the protein ^13^. We demonstrate herein the potential for albumin subjected to thermal or mechanical force to form supramolecular complexes with unfunctionalized ECM polymers and drastically change their mechanical or topological properties. Heilshorn and colleagues previously reported on supramolecular hydrogels formed *via* protein interactions with polysaccharide backbones ^14– 16^. In the present work we exploit interactions between BSA and GAG polymers to generate structurally diverse protein-glycosaminoglycans complexes. We examine how these negatively charged biopolymers interact and self-assemble with BSA, and develop non-covalent and covalent systems formed from HA/BSA, HA/CS, and CS/BSA interactions.

## Methods

### Chemicals and reagents

All starting materials were purchased from Sigma Aldrich and used as received unless stated otherwise.

### Electrostatic surface potential modeling

The crystal structure of bovine serum albumin (BSA) was downloaded from Protein Data Bank (ID:3V03). The second chain of the homodimer in the crystal structure was removed to display BSA in monomeric form. Using the Adaptive Poisson-Boltzmann Solver (APBS) tool available through PyMOL v2.2.2, the electrostatic surface potential was calculated under default parameters ^17^. The section of the protein surface showing the previously-described GAG binding pocket was rendered with PyMOL.

### Particle formation

A 10 wt% chondroitin sulfate (C9819 Sigma) solution was prepared by mixing the polymer powder in Milli-Q H _2_O (18 mΩ) at room temperature overnight. Bovine serum albumin (BSA; 50 mg/mL; A3294 Sigma) was added to the solution and rigorously mixed for 12 h (1000 RPM) at room temperature. Lower total concentrations of polymer and protein took more than 12 h to form particles.

### Gel formation

4 wt% hyaluronic acid (1.5–1.8 MDa; 53747 Sigma) solutions were prepared by mixing the polymer powder in Milli-Q H _2_O (18 mΩ) at 40 °C for 40 h. The solution was sealed and stored at 4 °C until further use. To electrostatically crosslink HA, BSA (A3294 Sigma) solutions were first heated to 80 °C for 2 h. The resulting solutions were mixed with HA solutions at room temperature and mixed rigorously at 1000 RPM until homogeneous, typically 20 min. The BSA concentration was varied but the HA concentration for HA/BSA systems was at 1 wt%. HA/BSA samples with heat treated albumin were observed to be opaque, whereas blends with unheated protein were transparent. It was observed that unheated BSA would not result in gel formation (see results and discussion). To form HA/CS/BSA blends, dry chondroitin sulfate (10 wt%) and dry BSA were added to HA solutions and mixed vigorously with a metal spatula. Poly(caprolactone) (PCL) blends were formed by mixing poly(caprolactone) diol (M *_n_* = 2 kDa; Sigma 189421; 5 wt%) with poly(caprolactone) diol melt (M *_n_* = 550 Da; Sigma 189405) and mixing at 700 RPM at 50 °C overnight. A specified amount of rhodamine B was added to the blend for ultraviolet–visible spectroscopy (UV-Vis). The covalent HA/BSA system used for SEM studies was formed by heating all components together to 80 °C for 2 h.

### Rheology

Rheological sweeps were conducted on an Discovery HR-2 Rheometer (TA Instruments, New Castle, DE, USA) with 8 mm, 20 mm, or 40 mm geometries all at 20 °C Zero gap, rotational mapping (precision bearing mapping; 2 iterations), geometrical inertia, and friction calibrations were done prior to each use of the rheometer. Samples were loaded onto the rheometer with a 600–1000 *µ*m loading gap. A water trap was placed to prevent dehydration. Amplitude sweeps were conducted, usually at 10 rad/s, to determine the strains within the linear viscoelastic region.

### Dynamic light scattering

Dynamic light scattering (DLS) measurements were carried out on a Malvern Zetasizer NS90 instrument at room temperature and standard settings. Samples were analysed in a 1.5 mL PS cuvette (Fisher Brand).

### Transmission electron microscopy

Transmission electron microscopy (TEM) was carried out on a FEI Philips Tecnai 20. Samples were prepared on holey carbon grids by pipetting 1 *µ*L of desired aqueous solution and allowing it to evaporate under ambient conditions (drop-casting). Particle size distributions were calculated by counting the diameters of more than 100 particles.

### Scanning electron microscopy

Scanning electron microscopy (SEM) was carried out on a Tescan MIRA3 SEM. Samples were freeze dried in small volumes and thin flakes were carefully mounted onto sample stubs covered in carbon conductive adhesive tapes. Each sample was then placed into a platinum sputtering system and sputter-coated to 10 nm thickness. To load the sample, the SEM chamber was vented and the sample stubs were loaded and tightened into places. After closing the sample compartment and allowing the system to reach vacuum, images were captured using operating voltage ranging from 10–30 kV.

### UV-Vis Spectroscopy

UV-Vis spectroscopy was performed using a Mikropack DH-2000 UV-Vis-NIR Halogen light source and an OceanOptics USB2000 Fiber Optic Spectrometer. Spectra from 375 nm to 750 nm were recorded at 150 ms integration time and time intervals of 60 s.

### Compiling brain glycosaminoglycan measurements

To estimate brain chondroitin sulfate (CS) levels, all articles cited as using the Blyscan assay to measure sulfated GAGs were searched against the keywords “brain” and “neural”. A total of 7 articles were found meeting the criteria of measuring sulfated GAGs in mammalian brain tissue. A separate literature search of hyaluronic acid (HA) measurements identified 2 articles. Reported concentration values were converted to molarities, representing the moles of disaccharide repeat units per volume of native brain tissue, assuming a brain density of 1.04 g/mL ^18^. In cases where brain weight was reported as dry mass instead of native tissue, masses were converted by assuming that 77% of brain weight is water ^19^.

## Results and discussion

In this work we explored the self-assembly of charged proteins with negatively-charged polysaccharides endogenous to the human brain. To facilitate supramolecular crosslinking of these polysaccharides, we introduced BSA, which has electrostatic binding pockets complementary to anionic GAGs, analogous to the binding interfaces of ECM proteins.

**Figure 2.**
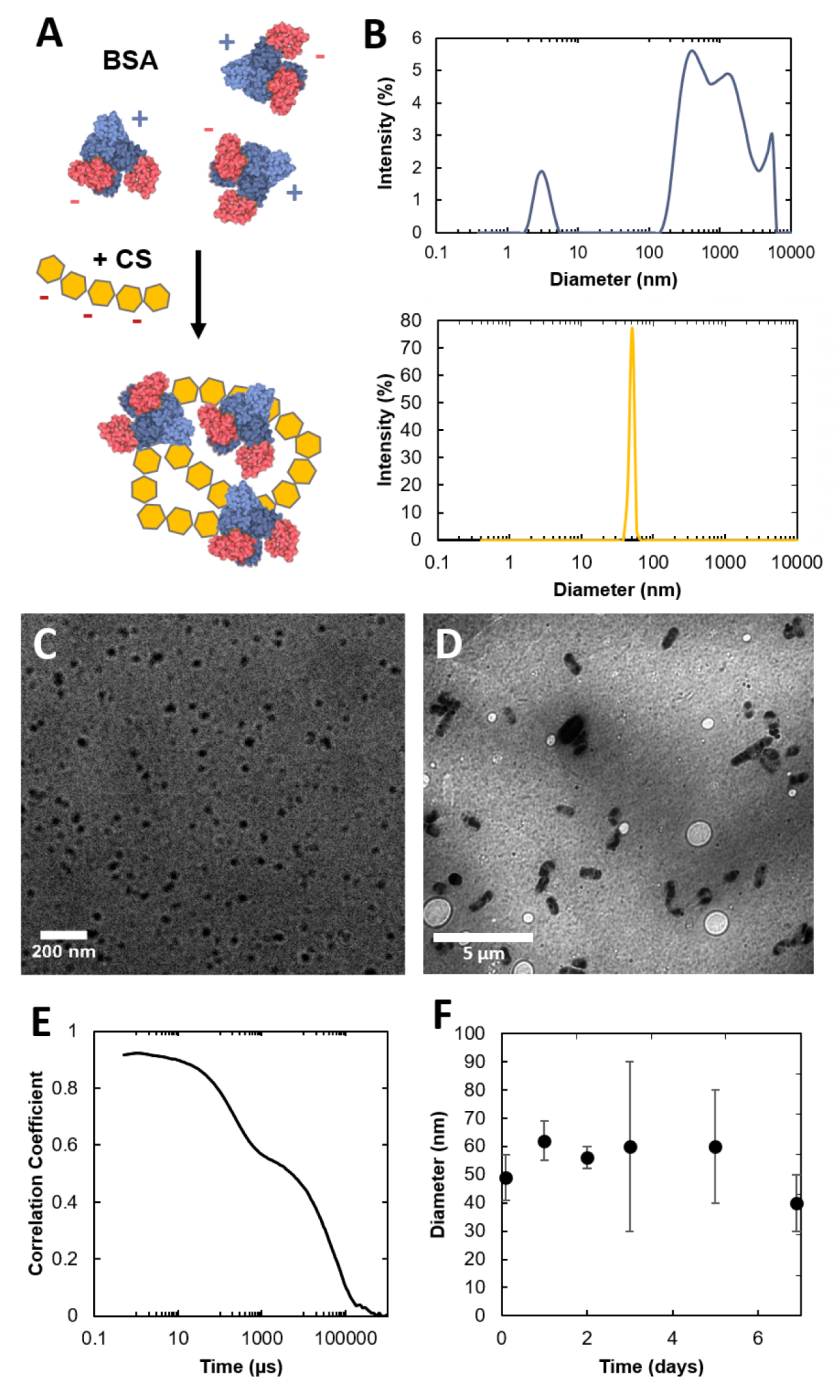
Self-assembly of chondroitin sulfate (CS) and bovine serum albumin (BSA) particles. ( **A**) Schematic of the formation of dense CS-BSA particles. ( **B**) DLS size plot of dynamic BSA aggregates (top) and CSBSA particles (bottom). ( **C**– **D**) TEM image of CS-BSA nanoparticles ( **C**) and microparticles ( **D**). ( **E**) Autocorrelation function of CS/BSA NPs. ( **F**) Time resolved DLS of CS/BSA NPs. Dataset 1: Dynamic light scattering and transmission electron microscopy data ^20^.

BSA and CS polymer solutions were mixed rigorously overnight and allowed to self-assemble into nanostructures. Two distinct populations of particles were observed to form ( Figure 2B–D, Dataset 1 ^20^), and were dissimilar to the flocculation of BSA aggregates alone. The smaller particles were characterized with dynamic light scattering (DLS) and transmission electron microscopy (TEM). DLS yielded an average particle diameter (D) of 51 *±* 3 nm. These structures were stable for at least 2 days in the parent suspension ( Figure 2E). DLS autocorrelation data suggested that a large diameter species may also be present in the solution ( Figure 2F). TEM was used to characterize these self-assembled microparticles ( Figure 2D). The analysis of these micrographs indicated the presence of two distinct populations of assembles, with the mean diameter of D = 60 *±* 10 nm, that is consistent with DLS experiments, and an additional one of D = 1.5 *±* 0.5 *µ*m. In the brain, CS is covalently scaffolded onto protein cores (e.g. aggrecan ^21^) and binds with many ECM proteins through non-covalent, supramolecular interactions ^22^. The non-covalent interactions between CS and BSA described here, potentially owed to electrostatic interactions or phase separation, could provide insight into driving forces that contributes to GAG aggregation and nanostructure formation *in vivo*.

**Figure 3.**
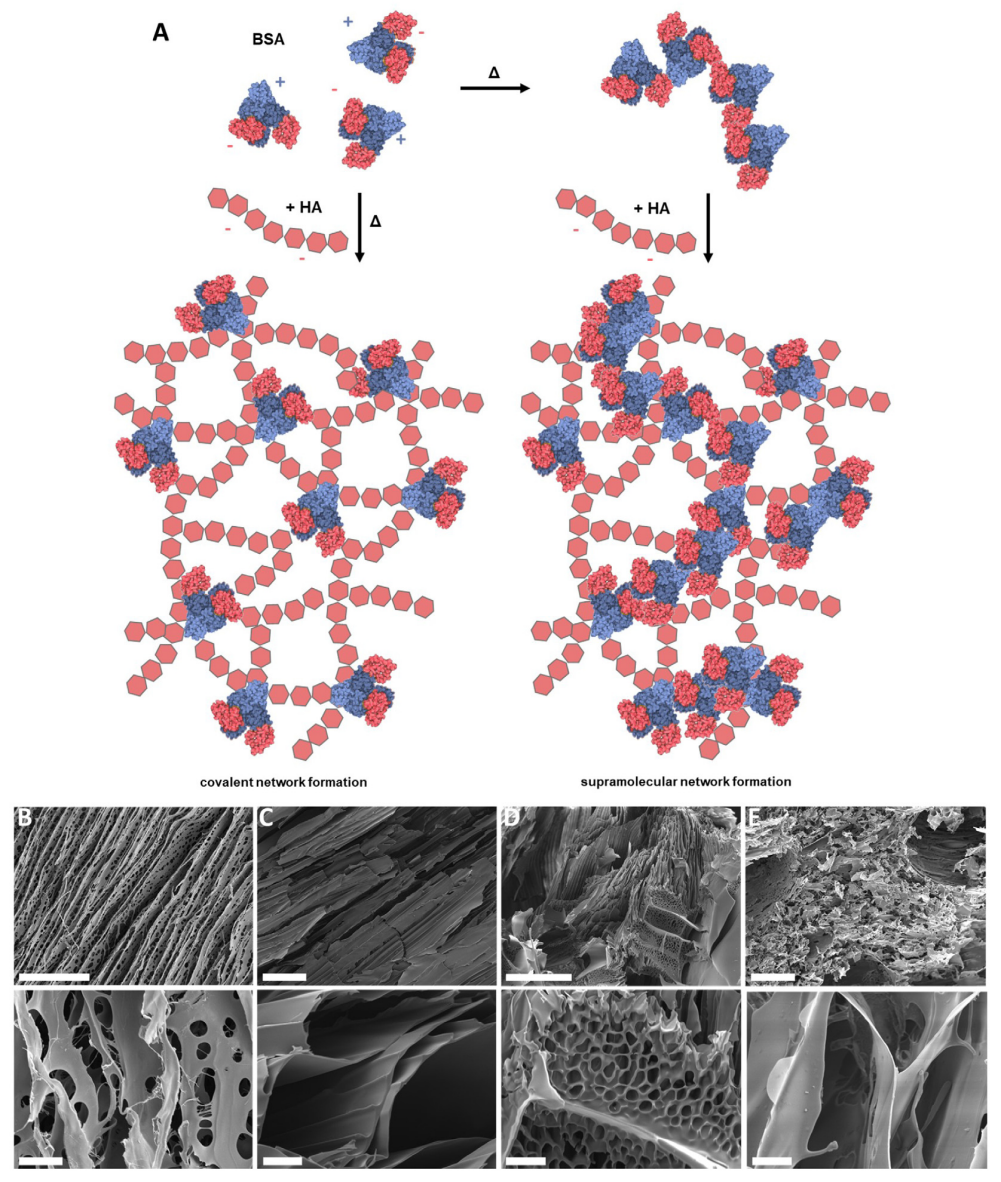
HA/BSA gels. ( **A**) Illustration of strategies to form gels from BSA and HA. Changing the order of addition and protein concentration allows facile tuning of the mechanical and topological properties of the network. ( **B**– **E**) SEM images of HA alone, HA/BSA (untreated and undenatured; 1:5), HA/BSA covalent gels ^23^ (1:5) which are not otherwise considered in this study, and supramolecular HA/BSA gels (1:5; see Figure 4). Scale bar = 100 *µ*m (top), 10 *µ*m (bottom). Dataset 1: Scanning electron microscopy data.

**Figure 4.**
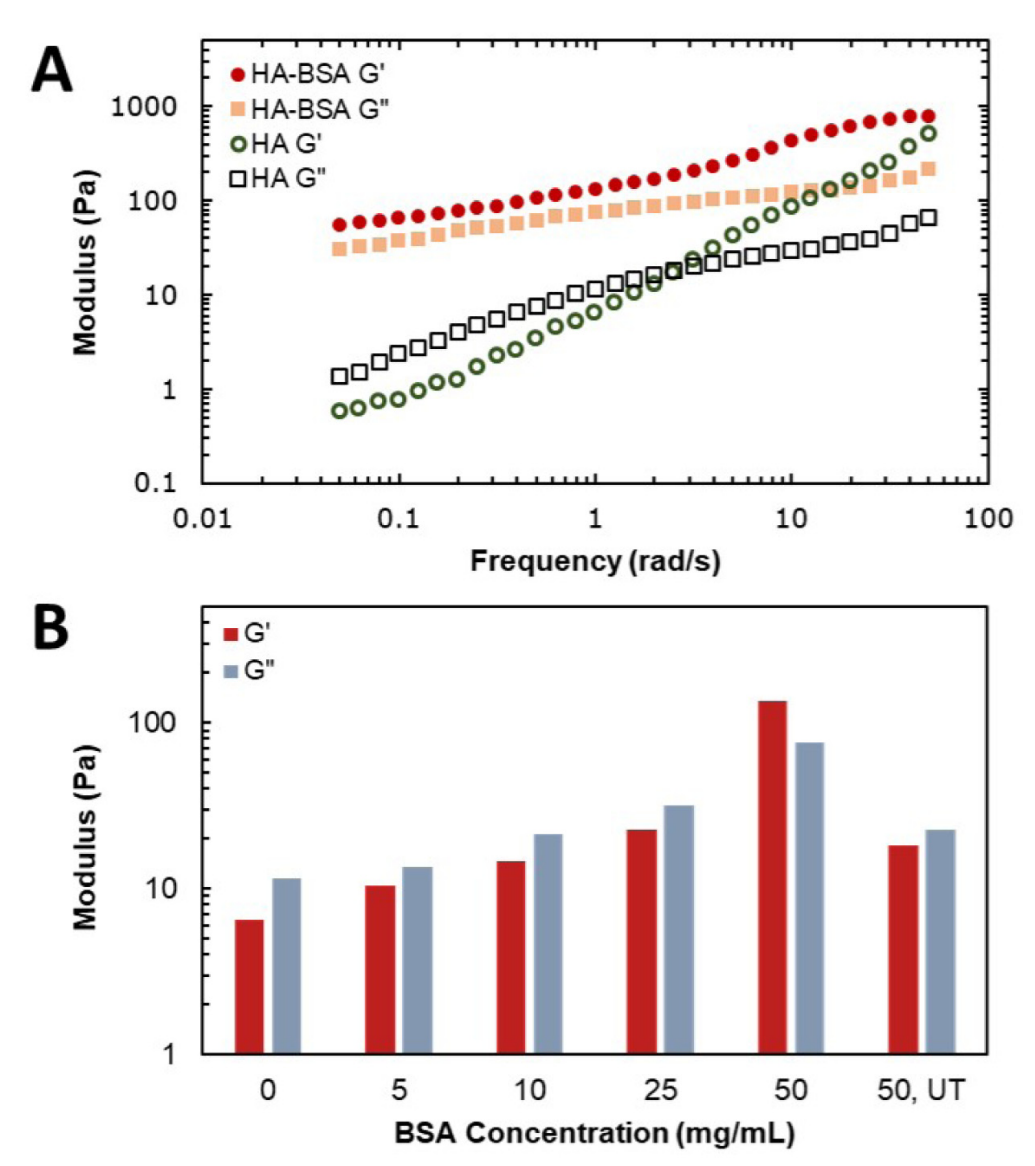
Rheological characterization of HA/BSA systems. ( **A**) Oscillatory rheological frequency sweep of HA solution alone (10 mg/mL) and HA/BSA gel (BSA: 50 mg/mL). ( **B**) Storage (G’) and loss (G”) moduli of HA (10 mg/mL) with various concentrations of BSA at 1 rad/s. UT = untreated BSA. Dataset 1: Rheology data.

We then turned our attention to HA, a linear high molecular weight polysaccharide that is the only supramolecular GAG in human physiology. HA and heat treated BSA were mixed and the interactions between the charged moieties of the GAG and the albumin solution led to the self-assembly of a supramolecular gel ( Figure 3– Figure 4, Dataset 1 ^20^). Solutions of HA alone were viscous but did not show gel-like properties. Oscillatory rheological measurements were used to probe the mechanical properties of these materials. Introduction of BSA resulted in the formation of a gel with stiffness comparable to central nervous system tissue ^24^. It was observed that the same concentration of BSA wihtout the heat treatment did not result in gelation ( Figure 4B). This is perhaps owed to the fact that in its folded form, the BSA protein has a singular charged binding pocket that cannot facilitate multi-chain interactions ( Figure 1B), and thus cannot serve as an effective crosslinker. Heat-treated BSA, however, shows greater affinity towards multi-chain interactions and crosslinks the system to form a physical gel. The differences in topology between these systems can be seen further in scanning electron microscopy (SEM) images ( Figure 3B–E). The covalent and supramolecular systems differ from HA with non-treated BSA. The underlying structural changes that occurs when this solution of BSA is heated remains to be elucidated and is exciting future work.

**Figure 5.**
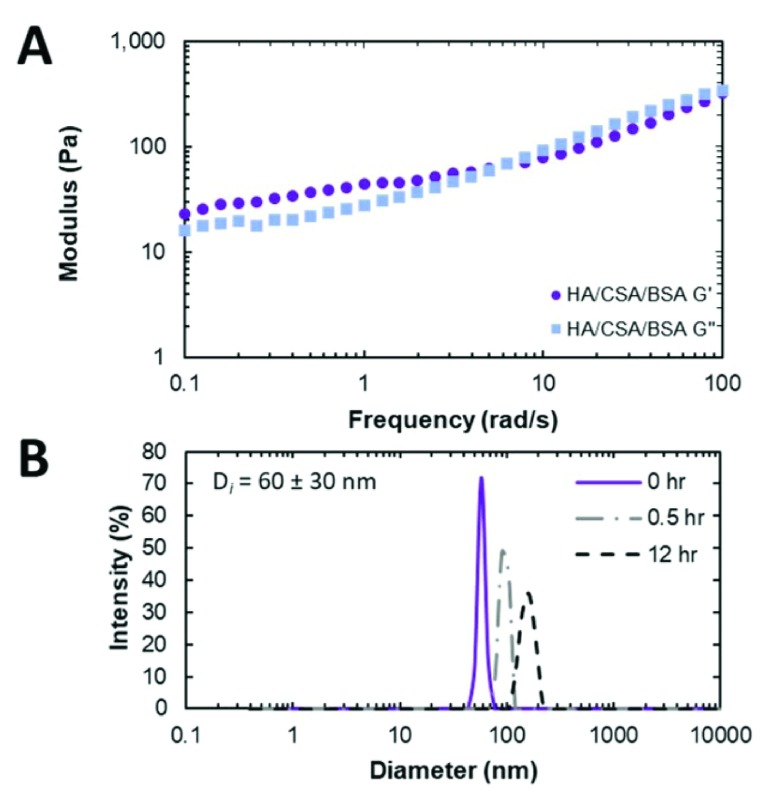
Blend characterization. ( **A**) Oscillatory frequency sweeps of protein-polysaccharide blend. ( **B**) Qualitative time-resolved dynamic light scattering experiment of the sheared blend. Dataset 1: Rheology and dynamic light scattering data.

**Figure 6.**
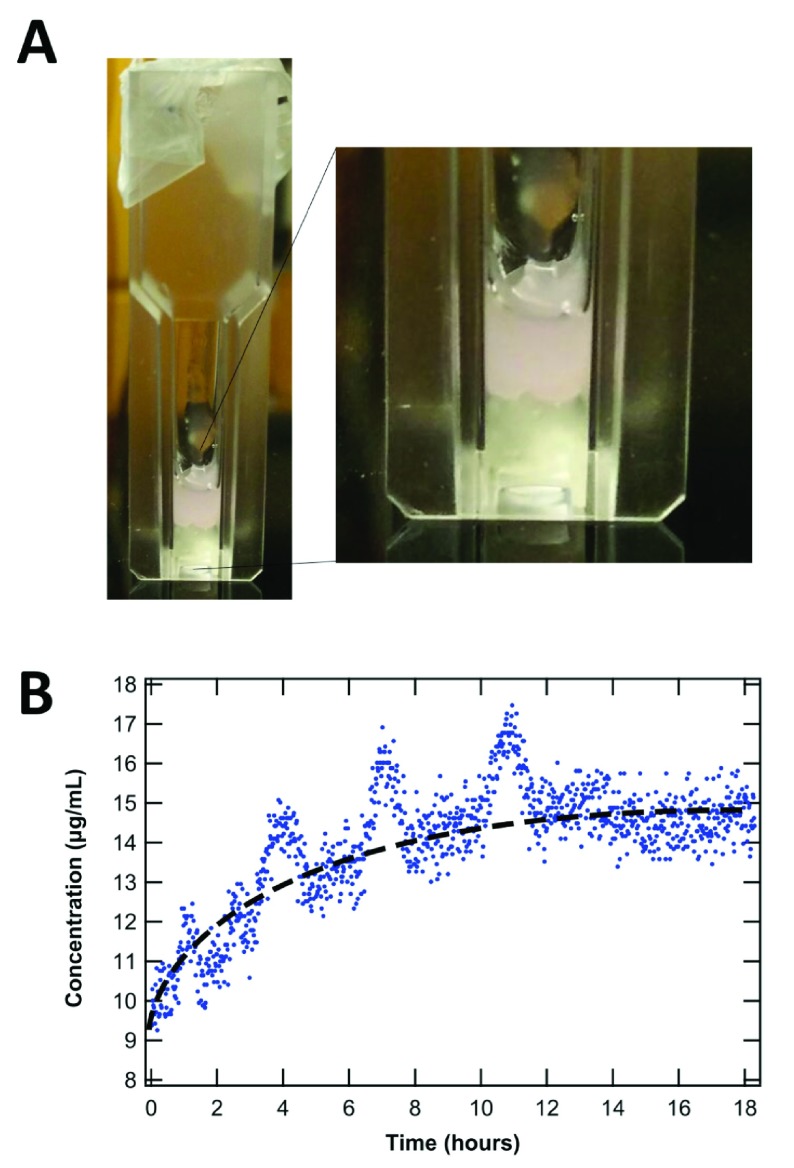
Measuring interfacial mass transfer of a small molecule between hydrophilic and hydrophobic phases. ( **A**) Picture of PCL loaded with rhodamine (pink) on top of the HA/CS/BSA blend system, representing the hydrophobic and hydrophilic interface for potential modeling of mass transfer in drug delivery *via* UV-Vis spectroscopy. ( **B**) Concentration at interface after large bolus release over time. Hydrophobic phase drug concentration = 10 *µ*g/mL. Average hydrophilic phase equilibrium concentration = 14.5 *µ*g/mL. The periodic fluctuations in concentration were attributed to trapped air interfering with the interface. Dataset 1: UV-Vis data.

We then studied a HA/CS/BSA blend generated *via* mechanical force, similar to the CS/BSA particle system. DLS data qualitatively showed that when combined with these charged polysaccharides, BSA exhibited different behavior than for the protein alone or the protein with CS alone ( Figure 2, Figure 5). These studies suggest that the presence and composition of polymers in a protein-polymer mixture change albumin’s behavior. We explored whether these HA/CS/BSA systems could be used to study transport phenomena of the model drug rhodamine B (rhodB). Many parenteral drug-delivery studies monitor *in vitro* release kinetics into saline as a model for *in vivo* release. Here we explore the potential for this blend to replace saline and to monitor diffusion across an interface ( Figure 6). A hydrophobic blend of poly(caprolactone) (PCL) chains at different molecular weights loaded with rhodB was carefully added on top of the hydrophilic blend. We found that it was possible to monitor the interfacial concentration of rhodB with UV-Vis spectroscopy. Interestingly, we observed a large bolus release of the water-soluble drug from the hydrophobic phase to the hydrophilic phase at the interface until the same concentration was reached, subsequently an equilibrium or pseudosteady-state concentration was reached after 14 h. Such a system is potentially useful in modeling mass transfer of drugs between hydrophobic drug delivery materials into hydrophilic physiological conditions ^25^.

**Figure 7.**
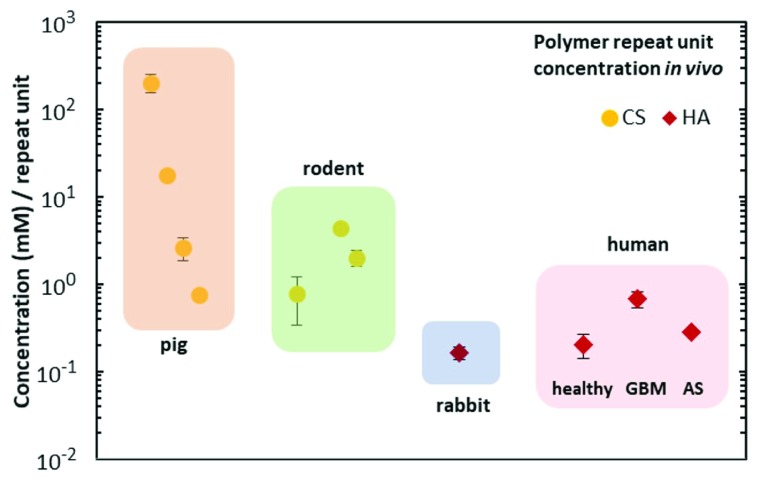
Summary of concentrations of hyaluronic acid (HA) and chondroitin sulfate (CS) in the brain of various species. The concentration of HA in healthy tissue, glioblastoma, and astrocytoma is also plotted.

Finally, prior tissue engineering studies have largely neglected the question of how the exact composition of the brain ECM might inform efforts to create biologically-mimetic hydrogels. To estimate the physiological concentrations of CS and HA that occur *in vivo* ( Figure 7, Dataset 1 ^20^), we summarize the literature for CS and HA measurements of mammalian brain tissue ^26– 33^. These estimates clustered in the millimolar range for CS disaccharides, and 100 micromolar range for HA.

## Conclusions

In this work we characterize non-covalent interactions between HA, CS, and BSA. We report nano- and microparticle self-assembly as well as gelation and supramolecular network formation. We also report on the mass transfer of a model drug from a hydrophobic phase into a proteinpolysaccharide hydrophilic phase, and suggest this approach may be more useful *in vitro* approach than measuring drug release into saline. Finally, we summarize reported concentrations of CNS HA and CS in different animal models and humans, which may be important in the design of matrices for CNS tissue engineering. This work offers a robust method of forming hydrogels from unfunctionalized HA, and is attractive for its simplicity compared to those derived from chemically functionalizing the polysaccharide backbone.

## Data availability

Underlying data for this study is available from Open Science Framework

OSF: Dataset 1: Protein-mediated gelation and nanoscale assembly of unfunctionalized hyaluronic acid and chondroitin sulfate, https://doi.org/10.17605/OSF.IO/3BXQG ^20^

Data is available under a CC0 1.0 License
