# Supplementary material for: Protein-mediated gelation and nano-scale assembly of unfunctionalized hyaluronic acid and chondroitin sulfate
Source: F1000Res. 2019 Nov 8;7:1827. Originally published 2018 Nov 20. [Version 3] doi: 10.12688/f1000research.16929.3 (PMC6688722; doi:10.12688/f1000research.16929.3)
Supplement: Peer review history

# Peer review: Reviewer response for version 2

**DOI:** 10.5256/f1000research.20518.r51412

**Published:** 2019-08-08

**Authors:** Michelle A. Calabrese

**Affiliations:** 1 Department of Chemical Engineering and Materials Science, University of Minnesota, Minneapolis, MN, USA

The paper by Tabet and co-workers examines the interactions between a model protein, BSA, and hyaluronic acid (HA) and/or chondroitin sulfate (CS) in forming self-assembled networks using rheology, DLS, imaging, and other techniques. The work is of a generally high standard and is suitable for indexing, but several points (below) should be addressed to improve the quality of the paper.

**Rheology measurements:**

 Why does the gap range between 600 um and 1 mm? The text says “amplitude sweep” was conducted – at what frequencies were they done or was just one done?

**DLS measurements:**

 What is the solution concentration used here?  Interactions between particles, especially electrostatic interactions, can cause two modes to appear in the DLS data; these do not necessarily correspond to sizes. As such, it is important to specify the concentration in the materials and methods section, and what interactions are expected. One way to check if the additional peaks are from interactions or not is to further dilute the samples. Additionally, what model used to produce the radii listed? Especially in cases where electrostatic interactions may be significant, the extrapolation of the data to “radius” may not be accurate; this should be considered by the authors.

 The analysis of the DLS data between Fig. 2-5 is also a bit confusing. How are we to interpret DLS data on a gel like this? Again, the radius/modeling assumptions seem like a stretch here so that should be stated… Also in Figure 5, could the larger sizes be coming out of aggregation that’s occurring/changes to structure that are occurring due to any interfaces in the cuvette? Because the effect is so slow/fairly minor, and it’s hard to really understand the DLS from a gel like this to start with, I think this interpretation should be re-examined.

 What makes something “more dynamic” (page 6 second to last paragraph). Based on the labelling, it seems as though both the gels (Fig. 5) and the particles (Fig. 4b) change over a 12h time scale so I’m not sure what more dynamic means. The authors state that the width of the distribution in the HA/BSA is why you assume there are not discrete particles because there is just one peak in the time zero data.

**Figure 3**:

c) At what frequency is this conducted? More detail is needed. Do you know that the change in modulus with the time sweep is real vs. can be attributed to drying? A water trap will not necessarily stop this over the 5 hour experiment course. What happens if you put this in a small Couette? Do you get a visual change if you leave these in a vial over time in terms of the flowability/pourability? Have you tried the measurements with a light oil or similar around the sample edges (not in the trap)?
The experiment that part c) is done after is not clear. Was the solution simply sheared to 100% (in which case, include the rate at which this was done)? Or was it oscillatory shear to 100% at a particular frequency? These details should be specified.
e) at what frequency is this comparison done at?

 

**Minor points:**

 With respect to the rheology methods section, “to determine a strain in…” should be “to determine the strains within” since multiple strains are within the LVE window.

 The legend in Fig. 3b,c is hard to differentiate between in black and white. While most rheologists will know which moduli is which, just based on symbol/coloration, it is difficult to tell which is G’ and G’’ in the key. I think it would help if G’ was open circles and G’’ was open squares for HA and have the same with closed symbols for HA+BSA.

I confirm that I have read this submission and believe that I have an appropriate level of expertise to confirm that it is of an acceptable scientific standard.

# Peer review: Reviewer response for version 2

**DOI:** 10.5256/f1000research.20518.r47121

**Published:** 2019-04-15

**Authors:** Laura De Laporte

**Affiliations:** 1 DWI − Leibniz-Institute for Interactive Materials, Aachen, Germany

No further comments.

I confirm that I have read this submission and believe that I have an appropriate level of expertise to confirm that it is of an acceptable scientific standard.

# Peer review: Reviewer response for version 1

**DOI:** 10.5256/f1000research.18510.r42740

**Published:** 2019-02-12

**Authors:** Jessica CF. Kwok

**Affiliations:** 1 School of Biomedical Sciences, Faculty of Biological Sciences, University of Leeds, Leeds, UK; 2 Institute of Experimental Medicine, CAS, Prague, Czech Republic

The article ‘Supramolecular protein-mediated assembly of brain extracellular matrix glycans’ by Tabet et al., describes the self-assembly of glycosaminoglycans hyaluronan (HA) and chondroitin sulfate (CS) in the presence of bovine serum albumin (BSA). The authors report that HA/BSA forms hydrogel upon mixing while CS/BSA forms nano- and micro-particles.

 The recognition of supramolecular assembly with biological materials has increased our understanding of how biological systems function in the last years. Here, the authors aim to address how HA and CS self-assemble for their function, emphasising on their role in the central nervous system. However, the study appears partial and the hypothesis is not clear.

 Hypothesis is not clear: Why do you use serum albumin? Serum albumin is not normally present in the brain tissue but confines inside the blood vessels in the brain. The penetration of serum albumin to the brain is protected by the blood-brain barrier. Extravasation of albumin to the brain will only happen in pathological conditions. This means that serum albumin is not the normal contributor to brain extracellular matrix (ECM) glycan assembly. This needs to be stated clearly in the article. Resulting from this, the title is also misleading, because the model described is thus not related to brain ECM.

 Please define clearly the meaning of ‘supramolecular’ in the article. In both the Abstract and the Introduction, the authors claimed that HA is the ‘only supramolecular glycosaminoglycan’. What do you mean by this? However, in the main text, the authors described CS/albumin interaction as ‘supramolecular interactions’. Please define clearly the meaning of the term.

 Regarding the CS/albumin interaction, the authors attribute particle formation from mixing CS with serum albumin is due to supramolecular interactions between CS and BSA. How can you rule out that this is not due to phase separation of your materials instead? Are the nano- and micro-particles stable over time?

 Regarding the model of drug release, the authors used rhodamine by laying it on top of the hydrogel. This is showing diffusion of molecules through the gel, but not the release of rhodamine from the gel. Please correct the terminology accordingly.

 Regarding the title, please change glycans to glycosaminoglycans.

 Minor changes:

 In the abstract, the first sentence: HA is ‘a’ major component of ECM in the CNS, but not “the” major component. CS is present in higher concentration than HA in the CNS, which is also indicated by the analysis reported in Figure 5 in the article. Please correct the sentence.

 Figure 1: The CS shown in Figure 1A is chondroitin 6-sulfate, while the CS used in the experiment (Sigma C9819) is a chondroitin 4-sulfate. Could you please amend the figure to represent the structure of the CS used?

 Figure 1: The HA structure is wrong. HA is formed by disaccharide repeats of [GlcNAc-β1,4-GlcA-β1,3]. The figure shows a [GlcNAc-β1,4-GlcA-β1,4] linkage. Please correct this accordingly.

 Page 4, first paragraph in the result section: ‘CS is covalently scaffolded onto peptide cores (e.g. aggrecan)’. Please change peptide into protein. Aggrecan is a big protein with molecular weight larger than 250 kDa even without the attachment of glycosaminoglycan chains.

 Figure 3C: what are the three lines in Figure C? Please state clearly, either in the figure or in the legend.

 Page 5, I assume ‘HA/CS/CS’ systems actually mean ‘HA/CS/BSA’ systems?

I confirm that I have read this submission and believe that I have an appropriate level of expertise to confirm that it is of an acceptable scientific standard, however I have significant reservations, as outlined above.

# Peer review: Reviewer response for version 1

**DOI:** 10.5256/f1000research.18510.r40915

**Published:** 2018-12-03

**Authors:** Laura De Laporte

**Affiliations:** 1 DWI − Leibniz-Institute for Interactive Materials, Aachen, Germany

The paper looks at the interaction of natural GAG, such as HA and CS, with BSA as model protein. Overall the data is clear but some improvements can be made. 

 Are the particles formed with CS/BSA hard particles or nano, microgels? You can do AFM to check this, by measuring the swelling behavior in media and determining the Young's modulus.

 For the HA/BSA interaction, gels are formed. Here more analysis can be done. The rheometer data only shows one data point. The G' for multiple gels can be measured to do statistics. Ideally these measurements should also be compared with media as the salts will affect the mechanical properties of the gel. Also, the gels seem to be very stiff for these natural materials. You decrease the concentrations to check whether you can vary the stiffness. Nervous tissue is usually in the range below 1 kPa. The authors mention the gel is shear thinning but this is not proven with rheology. Even a video of the gel would be helpful to have an idea about its properties, like transparency, shear thinning properties via pipetting, and gelation. In addition, even though SEM may cause some artifacts, it will still give you an idea about the internal structure of these gels compared to other gels reported in the literature. In Figure 3C, there is not legend and unclear what the different conditions are. For Figure 3D, as you have a gel, how can you also have individual particles for DLS measurements? Please make a note about this in the text.

 Figure 4: As Rhodamine is fluorescent, you can show fluorescent images to demonstrate the diffusion of the molecule into the gel.

 Some minor comments to improve the text:

 Abstract:

- Self-assembly
- HA has been crosslinked covalently via other mechanisms than photo-catalyzed ^1^^,^^2^^,^^3^.

Introduction:

- The authors mention that HA has been made into many covalently modified derivatives. Do they mean that functional groups were covalently bound to the HA backbone to make derivatives, or that functionalized HA can covalently bind to other molecules to form drug delivery and TE materials. This is repetitive with a sentence later in the intro: 'HA is often chemically modified…….'

Methods:

- The GAG binding pocket of BSA is not shown in Figure 1B.
- BSA should only be written full once, after that just use the abbreviation.

I confirm that I have read this submission and believe that I have an appropriate level of expertise to confirm that it is of an acceptable scientific standard, however I have significant reservations, as outlined above.

## References

[1] : Charge-Controlled Synthetic Hyaluronan-Based Cell Matrices. *Molecules*.2018;23(4) : 10.3390/molecules23040769 10.3390/molecules23040769 29584672PMC6017843

[2] : Novel enzymatically cross-linked hyaluronan hydrogels support the formation of 3D neuronal networks. *Biomaterials*.99: 10.1016/j.biomaterials.2016.04.036 47-55 10.1016/j.biomaterials.2016.04.036 27209262

[3] : Hyaluronic Acid Hydrogels Formed in Situ by Transglutaminase-Catalyzed Reaction. *Biomacromolecules*.2016;17(5) : 10.1021/acs.biomac.5b01587 1553-1560 10.1021/acs.biomac.5b01587 27014785
